# Editorial: Advancing next-generation immunotherapy and biomarker discovery: pioneering the future of treatment strategies in solid tumors

**DOI:** 10.3389/fonc.2026.1975534

**Published:** 2026-09-08

**Authors:** Yee Peng Phoon, Lily Wang, Xue-Yan He

**Affiliations:** 1 Center for Immunotherapy and Precision Immuno-Oncology, Department of Cancer Sciences, Cleveland Clinic, Cleveland, OH, United States; 2 School of Medicine, Case Western Reserve University, Cleveland, OH, United States; 3 Department of Cell Biology and Physiology, Washington University School of Medicine, St. Louis, MO, United States

**Keywords:** artificial intelligence (AI), biomarker discovery, clinical translation & RCTs, next generation immunotherapy, personalized & precision medicine, precision immuno-oncology, resistance prevention, solid tumors

The evolution of cancer immunotherapy has fundamentally transformed the treatment landscape of solid tumors, creating unprecedented opportunities for durable responses and long-term survival. Immune checkpoint inhibitors (ICIs), adoptive cellular therapies, cancer vaccines, and emerging immunomodulatory approaches have demonstrated substantial clinical benefit across multiple malignancies. Despite these advances, significant challenges remain, including heterogeneous treatment responses, primary and acquired resistance, treatment-related toxicities, and a lack of robust biomarkers capable of reliably identifying patients who are most likely to benefit from immunotherapy. Addressing these limitations requires continued progress in both next-generation immunotherapeutic strategies and biomarker discovery. This Research Topic, *“Advancing Next-Generation Immunotherapy and Biomarker Discovery: Pioneering the Future of Treatment Strategies in Solid Tumors,”* was established to showcase innovative research spanning basic immunology and clinical oncology. Its overarching goal is to advance novel, biomarker-driven therapeutic strategies for solid tumors, thereby accelerating the development of precision immuno-oncology.

This Research Topic comprises 11 articles, including original research studies, reviews, a clinical trial report, and a case report, covering key areas such as biomarker development, mechanisms of immune regulation and resistance, emerging therapeutic platforms, precision oncology, and global implementation challenges. Collectively, these contributions highlight the growing convergence of therapeutic innovation and biomarker-guided decision-making that is shaping the future of cancer care.

**Figure 1:**
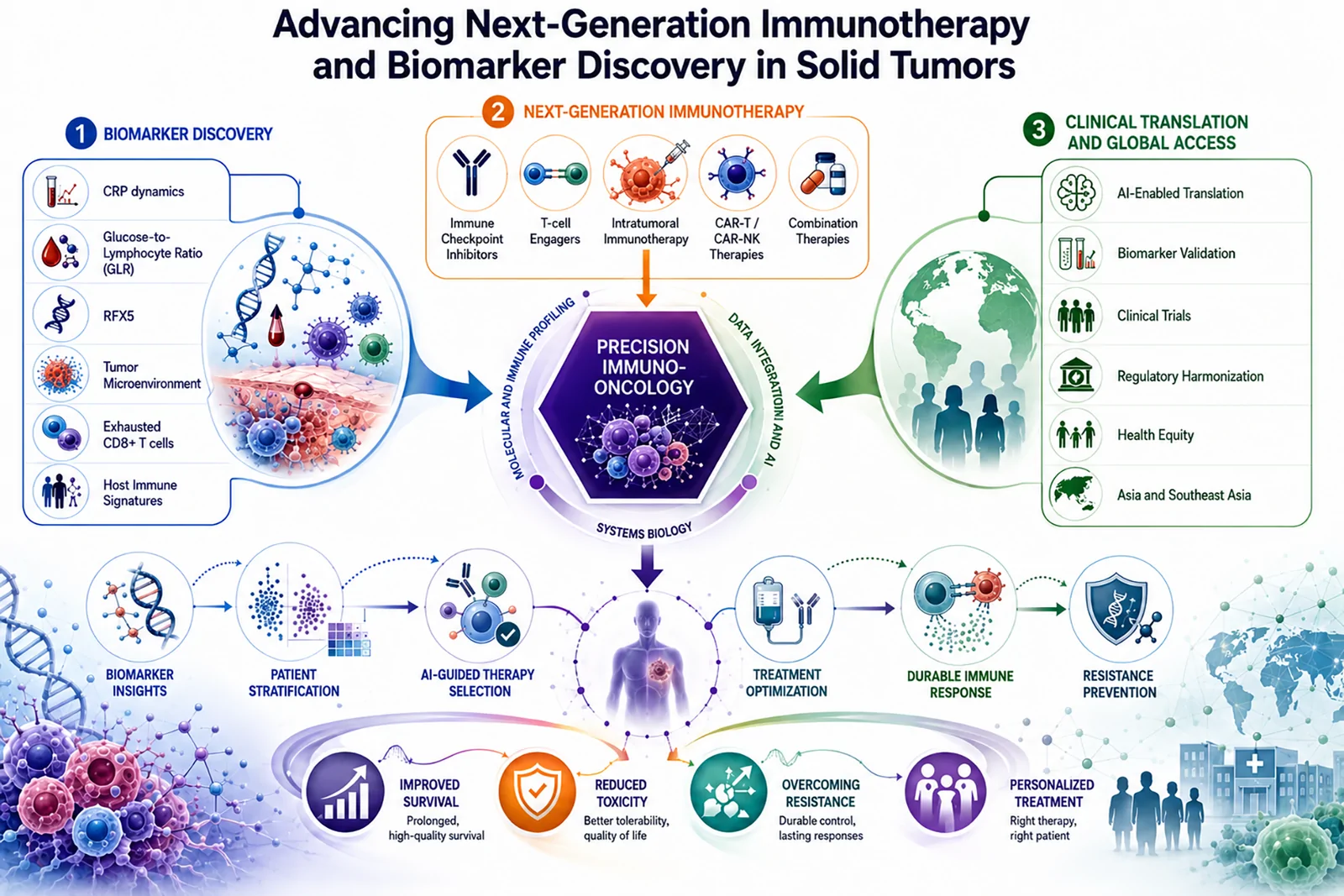
Roadmap toward precision immuno-oncology in solid tumors. Biomarker discovery and next-generation immunotherapeutic innovations converge through clinical translation, artificial intelligence, biomarker validation, and global implementation efforts to advance precision immuno-oncology and improve patient outcomes.

**Figure 1** summarizes the central themes of this Research Topic. The conceptual roadmap illustrates how advances in biomarker discovery, next-generation immunotherapies, and clinical translation are driving precision immuno-oncology. Emerging biomarkers, including inflammatory signatures, metabolic indices, host immune features, tumor microenvironment factors, and molecular determinants of immune responsiveness, are increasingly informing patient stratification and treatment selection. In parallel, novel therapeutic modalities such as ICIs, T-cell engagers, intratumoral immunotherapy, CAR-based cellular therapies, and combination strategies are broadening the therapeutic landscape. The integration of these advances with artificial intelligence (AI), biomarker validation, clinical trials, regulatory harmonization, and equitable implementation has the potential to improve outcomes, reduce toxicity, overcome resistance, and advance personalized treatment.

A central theme emerging from this Research Topic is the increasing importance of clinically accessible biomarkers. In patients with advanced non-small cell lung cancer receiving nivolumab, Ersoy et al. identified the baseline glucose-to-lymphocyte ratio (GLR) as a potential prognostic biomarker, supporting the relevance of systemic metabolic and inflammatory parameters in determining immunotherapy outcomes. Similarly, Wang et al. demonstrated that early reductions in C-reactive protein (CRP) levels were associated with improved progression-free and overall survival in patients with extensive-stage small cell lung cancer who were treated with adebrelimab-based chemoimmunotherapy. Together, these studies suggest that dynamic inflammatory biomarkers may provide practical and cost-effective tools for treatment monitoring and patient stratification.

Biomarker development also extends to toxicity prediction. Guo et al. investigated blood-derived inflammatory indices associated with immune checkpoint inhibitor-related cardiotoxicity and identified markers that may help predict disease severity and prognosis. As immune-related adverse events increasingly influence treatment selection and management, predictive biomarkers are becoming an essential component of precision immunotherapy.

Several studies focused on the biological mechanisms underlying tumor immunity and therapeutic responsiveness. Zhang et al. reviewed the multifaceted role of regulatory factor X5 (RFX5) in tumor biology, antigen presentation, and immune regulation, highlighting its potential as both a biomarker and a therapeutic target. Similarly, Guo et al. reviewed terminally exhausted CD8+ T cells in solid tumors, emphasizing their biological significance and potential applications as biomarkers and therapeutic targets. These studies underscore the importance of understanding immune regulatory pathways and cellular states that influence treatment outcomes.

Beyond biomarker discovery, this Research Topic highlights advances in next-generation immunotherapy. For example, Wu et al. reviewed the rapidly expanding field of T-cell engagers, discussing their mechanisms of action, clinical development, and growing relevance in solid tumors. Although these agents have achieved considerable success in hematologic malignancies, their application to solid tumors may help overcome existing therapeutic limitations and broaden the impact of immune-based therapies.

Novel approaches to immune checkpoint blockade were evaluated by Tan et al., who investigated the intratumoral administration of PD-1/PD-L1 and CTLA-4 inhibitors in patients with recurrent or refractory solid tumors. Their study demonstrates the feasibility of localized immune modulation and suggests a promising strategy for the enhancement of antitumor activity while potentially minimizing systemic toxicity. These approaches may contribute to overcoming resistance and expanding treatment options for patients with advanced cancers.

The importance of multimodal treatment strategies is reflected in additional clinical and translational studies. Peng et al. evaluated the BRICS sequential therapeutic regimen in patients with refractory proficient mismatch repair colorectal cancer, a population that has been traditionally resistant to checkpoint inhibition. Meanwhile, Liu et al. reported prolonged survival in a patient with four synchronous primary malignancies who was treated with a multimodal regimen incorporating envafolimab, chemotherapy, and radiotherapy. These studies illustrate the ongoing shift toward individualized combination therapies designed to optimize antitumor immunity and address disease-specific challenges.

The need for broader and more integrated biomarker frameworks was highlighted in the review by Ismaili on gastric cancer immunotherapy. The author argued that future biomarker development should move beyond tumor-centric approaches to incorporate host immune factors, systemic inflammation, and tumor microenvironment characteristics. This perspective reflects a recurring theme throughout the Research Topic: successful precision immunotherapy will likely require multidimensional biomarker models capable of capturing the complexity of tumor-host interactions.

A distinctive contribution is the review by Bustamam and Phoon, which extends the discussion from scientific innovation to real-world implementation across Asia and Southeast Asia. The authors described advances in ICIs, CAR-T therapies, biomarker-guided precision medicine, and emerging technologies while highlighting barriers that limit wider adoption, including healthcare disparities, inadequate biomarker validation, restricted access to advanced therapies, underrepresentation in clinical trials, and regulatory challenges. The review further explored opportunities involving AI, telemedicine, international collaboration, and regulatory harmonization that could improve access to personalized cancer care. Importantly, it emphasizes that realizing the full potential of immunotherapy requires not only scientific progress but also equitable implementation across diverse healthcare systems.

Taken together, the contributions in this Research Topic identify several priorities for the future of precision immuno-oncology. Emerging biomarkers require prospective validation and integration into clinically actionable models to enable more precise patient selection and treatment decisions. Advances in single-cell sequencing, spatial transcriptomics, proteomics, and multi-omics technologies offer unprecedented opportunities to elucidate mechanisms of resistance and identify new therapeutic targets. Continued innovation in T-cell engagers, engineered cellular therapies, intratumoral interventions, and multimodal treatment approaches is essential to expanding the benefits of immunotherapy to a broader patient population. Equally important is the need to address disparities in access to immunotherapies, diagnostics, biomarker testing, and clinical trials through international collaboration, policy innovation, and regulatory harmonization. Together, these efforts are essential for translating scientific advances into accessible, effective, and personalized cancer care.

Ultimately, the future of precision immuno-oncology depends on the successful integration of biological discovery, biomarker-guided patient selection, therapeutic innovation, and equitable healthcare implementation. As these fields continue to converge, the vision of delivering the right immunotherapy to the right patient at the right time is becoming increasingly attainable, with the potential to transform cancer care on a global scale.

